# The impaired distribution of adenosine deaminase isoenzymes in multiple sclerosis plasma and cerebrospinal fluid

**DOI:** 10.3389/fnmol.2022.998023

**Published:** 2022-09-20

**Authors:** Barbara Kutryb-Zajac, Ada Kawecka, Fionä Caratis, Krzysztof Urbanowicz, Alicja Braczko, Tomomi Furihata, Bartosz Karaszewski, Ryszard T. Smolenski, Aleksandra Rutkowska

**Affiliations:** ^1^Department of Biochemistry, Medical University of Gdańsk, Gdańsk, Poland; ^2^Department of Anatomy and Neurobiology, Medical University of Gdańsk, Gdańsk, Poland; ^3^Laboratory of Clinical Pharmacy and Experimental Therapeutics, School of Pharmacy, Tokyo University of Pharmacy and Life Sciences, Hachioji, Japan; ^4^Department of Adult Neurology, Medical University of Gdańsk and University Clinical Center, Gdańsk, Poland

**Keywords:** adenosine deaminase (ADA), adenosine, multiple scleorsis (MS), endothelium, ADA1, ADA2, nucleotides

## Abstract

**Background:**

Adenosine deaminase (ADA) via two isoenzymes, ADA1 and ADA2, regulates intra- and extracellular adenosine concentrations by converting it to inosine. In the central nervous system (CNS), adenosine modulates the processes of neuroinflammation and demyelination that together play a critical role in the pathophysiology of multiple sclerosis (MS). Except for their catalytic activities, ADA isoenzymes display extra-enzymatic properties acting as an adhesion molecule or a growth factor.

**Aims:**

This study aimed to explore the distribution and activity of ADA1 and ADA2 in the plasma and the CSF of MS patients as well as in the human brain microvascular endothelial cells (HBMEC), human brain vascular pericytes and human astrocytes.

**Methods and results:**

The enzyme assay following reverse phase-high performance liquid chromatography (HPLC) analysis was used to detect the ADA1 and ADA2 activities and revealed an increased ratio of ADA1 to ADA2 in both the plasma and the CSF of MS patients. Plasma ADA1 activity was significantly induced in MS, while ADA2 was decreased in the CSF, but significance was not reached. The brain astrocytes, pericytes and endothelial cells revealed on their surface the activity of ADA1, with its basal level being five times higher in the endothelial cells than in the astrocytes or the pericytes. In turn, ADA2 activity was only observed in pericytes and endothelial cells. Stimulation of the cells with pro-inflammatory cytokines TNFα/IL17 for 18 h decreased intracellular nucleotide levels measured by HPLC only in pericytes. The treatment with TNFα/IL17 did not modulate cell-surface ATP and AMP hydrolysis nor adenosine deamination in pericytes or astrocytes. Whereas in endothelial cells it downregulated AMP hydrolysis and ADA2 activity and upregulated the ADA1, which reflects the ADA isoenzyme pattern observed here in the CSF of MS patients.

**Conclusion:**

In this study, we determined the impaired distribution of both ADA isoenzymes in the plasma and the CSF of patients with MS. The increased ADA1 to ADA2 ratio in the CSF and plasma may translate to unfavorable phenotype that triggers ADA1-mediated pro-inflammatory mechanisms and decreases ADA2-dependent neuroprotective and growth-promoting effects in MS.

## Introduction

Multiple sclerosis (MS) is a chronic inflammatory neurodegenerative disease of the brain and the spinal cord characterized by neuronal inflammation, demyelination and degeneration of axons. In MS, peripheral lymphocytes infiltrate the central nervous system (CNS) where they propagate inflammatory events by further recruitment of immune cells and activation of local glial cells (McFarland and Martin, 2007; Ortiz et al., 2014). The neurovascular unit, or the blood-brain barrier (BBB), broadly consists of the basement membrane, endothelial cells, pericytes and the astrocytic endfeet which together regulate the passage of molecules between the blood and the CNS. Dysfunction of the BBB occurs early and focally in MS and its increased permeability is a marker of MS-associated neuroinflammation (Cramer et al., 2015; Spencer et al., 2018).

Adenosine signaling in neurons and glial cells plays a significant role in neurological diseases due to its involvement in neurotransmission, neuromodulation, inflammation, regeneration, and regulation of BBB permeability to cells and molecules (Dunwiddie and Masino, 2001). Under pathological stimulation, cells extensively release ATP that promotes pro-inflammatory responses by interaction with purinergic receptors P2X and P2Y, while its breakdown product, adenosine, manifests anti-inflammatory and immunosuppressive activity (Domercq et al., 2019).

The metabolism of extracellular ATP is conducted by cell-surface ecto-enzymes, broadly expressed in the CNS (Zimmermann, 2008). These include ecto-nucleoside triphosphate diphosphohydrolase 1 (eNTPD1, CD39), which hydrolises adenosine triphosphate (ATP) via diphosphate (ADP) to monophosphate (AMP), and ecto-5’-nucleotidase (e5’NT, CD73) that dephoshorylates AMP to form adenosine. Adenosine deaminase (ADA) catalyzes the deamination of adenosine and deoxyadenosine into inosine and deoxyinosine, respectively. It maintains catalytic activity intracellularly, on the cell surface or as a soluble form in body fluids (Franco et al., 1997). These processes seem to be controlled by the release of cytokines on the cell surface after T cell activation (Cordero et al., 2001). Higher serum concentrations of inosine were observed in MS patients, and it has been concluded that the activity of soluble ADA increases in MS (Polachini et al., 2014). Similarly, ADA activity was enhanced in the cerebrospinal fluid (CSF) of MS patients in an isolated case report (Samuraki et al., 2017).

There are two isoenzymes of ADA, ADA1 and ADA2. ADA1 has a higher affinity to adenosine and neutral optimal pH. It is the most abundant in B and T lymphocytes and essential in the development of the acquired immune system. Its activity prevents the accumulation of toxic deoxyadenosine in proliferative cells and decreases the extracellular concentration of adenosine, which serves as an agonist for P1 receptors (Garcia-Gil et al., 2021). ADA1 also interacts with dipeptidyl peptidase-4 (CD26), causing the formation of ecto-ADA (eADA), and A1 and A2 adenosine receptors, expanding their activity in the striatum (Gracia et al., 2011). CD26 is more abundant in microglia and astrocytes than in neurons. Based on observation of CD26-ADA-A2AR complexes, it has been concluded that ADA may take part in the interaction between cells with CD26 antigen and those with adenosine receptors, including neurons (Moreno et al., 2018). Our recent studies revealed that also endothelial cells play a critical role as a source of ecto-ADA1 activity that was upregulated under endothelial activation and dysfunction (Kutryb-Zajac et al., 2016, 2019). This may be of special importance in the CNS, where microvascular endothelial-derived ADA can mute protective pathways dependent on adenosine receptor signaling (Bynoe et al., 2015). Although ADA2 deamination is less active than ADA1 due to lower affinity to adenosine, it may be crucial when adenosine concentrations are higher for instance in inflammatory conditions and tumorigenesis (Meyts and Aksentijevich, 2018). ADA2 is highly abundant in myeloid and microglial cells, and its shortage leads to vasculopathy, inflammation, hemorrhagic stroke, and various neurological disorders (Sozeri et al., 2021). Unlike ADA1, ADA2 does not interact with CD26, but it has been speculated that this isoenzyme can also bind to the cell surface and act as an ecto-enzyme (Zavialov et al., 2010). Moreover, it has been shown that soluble ADA2 is a dominant ADA isoenzyme in the human serum (Andreasyan et al., 2005). However, there is a lack of reports regarding the activity of ADA isoenzymes in MS patients. Therefore, this study aimed to analyze the distribution of ADA1 and ADA2 in the plasma and CSF of MS patients as well as determine these activities in the human brain microvascular endothelial cells (HBMEC), brain vascular pericytes and astrocytes.

## Materials and methods

### Human participants

Written informed consent was obtained from all patients in accordance with the Declaration of Helsinki, and the study was approved by the Independent Bioethics Committee For Scientific Research at the Medical University of Gdańsk, Poland under the license number: NKBBN/457/2019. Whole blood was collected from MS patients (*n* = 7, F/M = 7/0) at age 27 ± 4.0 (mean ± SEM) presenting with the clinically isolated syndrome (CIS) during a routine diagnostic procedure or non-MS patients (controls, *n* = 8, F/M = 6/2) at age 48 ± 9.7 (mean ± SEM). Cerebrospinal fluid (CSF) was collected from MS patients (*n* = 5, F/M = 4/1) at age 27 ± 4.0 (mean ± SEM) presenting with the CIS during a routine diagnostic procedure or non-MS patients (controls, *n* = 4, F/M = 3/1) at age 39 ± 8.4 (mean ± SEM) suspected of disorders requiring a lumbar puncture for diagnosis. Immediately after collection the CSF was centrifuged, aliquoted, and frozen at –80°C. Whole blood collected in plasma tubes was centrifuged, aliquoted, and stored at –80°C.

### Determination of the plasma and cerebrospinal fluid activities of adenosine deaminase iso-enzymes

To determine the activities of soluble total ADA (tADA) and ADA2 in plasma and CSF, 49 μL of each body fluid was pre-warmed to 37°C and incubated with adenosine (20 μM final concentration) in the presence or absence of ADA1 inhibitor, erythro-9-(2-hydroxy-3-nonyl) adenine (5 μM EHNA). After 30 min incubation, the reaction was stopped by deproteinization with 1.3 M HClO_4_ (ratio 1:1). The samples were maintained on ice for 15 min and centrifuged (20,800 × g, 4°C, 15 min). The supernatant was neutralized to pH 6.5–7.0 by 3 M K_3_PO_4_ and analyzed with HPLC-RP according to the modified method described earlier (Smolenski et al., 1990). Briefly, 2 μL of the sample was injected into a UHPLC system consisting of a Nexera LC40 set and an SPD-M30A diode array detector equipped with a high-sensitivity, 85 mm optical path cell (Shimadzu, Japan). Analytes were separated on a ReproSil-Pur 120 C18-AQ (150 × 2.0 mm ID, 4 μm) column with a dedicated guard (Dr. Maisch, Germany) using gradient elution at a flow rate of 500 μL/min. Column compartment temperature was set to 23°C. Mobile phase A consisted of 150 mM KCl and 150 mM phosphate buffer in ultrapure water, adjusted to pH 6 by controlling the ratio between mono- and dipotassium orthophosphate salts. Phase B was a 15% acetonitrile solution (v/v) of phase A. The gradient progression was as follows: 0 min, 0% B; 0.06 min, 0.5% B; 2.1 min, 2% B; 4.8 min, 22% B; 5.4 min, 100% B. The plateau was maintained for 1.2 min followed by 1.9 min of equilibration, resulting in a total analysis time of 8.5 min. Absorbance was monitored at 254 nm. Results were shown as μmol/min/L for plasma and nmol/min/L for CSF. The activity of ADA1 was determined by subtracting ADA2 activity from the total ADA.

### Cell culture and treatment

Conditionally immortalized HBMEC clone 18 (HBMEC/ci18), human brain vascular pericytes clone 37 (HBPC/ci37) and human astrocytes clone 35 (HASTR/ci35) originally created to build a tri-cell *in vitro* BBB model were used in this study in single-cell culture to investigate ADA expression and activity as well as the effects of TNFα/IL17 treatment on ADA activity. The astrocytes were grown in DMEM supplemented with 10% FBS, 1% N2 supplement-A (Stemcell, #07152), 1% penicillin/streptomycin and 4 μg/ml blasticidin S (for a detailed cell culture protocol see Furihata et al. (2016). Pericytes were grown in the pericytes growth media (ScienCell, #1201) supplemented with 4 μg/ml blasticidin S as described by Umehara et al. (2018). The endothelial cells were grown in EBM-2 BM (Cellab, CC-3162) (without gentamicin) supplemented with 1% penicillin/streptomycin, 10 mM GlutaMax and 4 μg/ml blasticidin S as described by Kamiichi et al. (2012). Endothelial cells were plated on collagen I (Merck, C3867-1VL) coated plates and all cells were plated in 24-well plates without blasticidin S. For treatment with TNFα/IL-17, cells were serum-starved for 2 h before treatments for 18 h with a mix of recombinant human TNFα (10 ng/ml) (Bio-techne, 210-TA) and recombinant human IL-17A (50 ng/ml) (Bio-techne, 317-ILB). All cells were grown and cultured at 33°C, 5% CO_2_.

### Determination of the rates of adenosine triphosphate and adenosine monophosphate hydrolysis and adenosine deaminase iso-enzymes’ activities in blood-brain barrier cells

To measure cell-surface ecto-enzymes’ activities, cells were incubated on 24-well plates with 1 ml of Hanks Balanced Salt Solution (HBSS). The rate of ATP hydrolysis and AMP hydrolysis were analyzed in the presence of 1 μM S-(4-Nitrobenzyl)-6-thioinosine (NBTI), nucleoside transporter inhibitor and 0.1 μM deoxycoformycin (ADA1 and ADA2 inhibitor) after the addition of 50 μM ATP or AMP. Total ADA activity was measured only in the presence of NBTI after the addition of 50 μM adenosine. Cell surface ADA2 activity was measured in the presence of adenosine and additional 5 μM EHNA (ADA1 inhibitor). After the addition of substrates for ecto-enzymes, samples were collected after 0, 5, 15, and 30 min of incubation at 37°C and analyzed using HPLC-RP as described above. The rate of nucleotide hydrolysis and adenosine deamination was calculated from a linear phase of the reaction and expressed per protein content as nmol/min/mg prot. Protein concentration was measured using the Bradford method.

### Determination of intracellular nucleotide concentration in blood-brain barrier cells

After 18 h treatment with 50 ng/ml IL17 and 10 ng/ml TNFα, the cell medium was removed from 24 well plates and the adherent cells were washed twice with HBSS. Then 300 μL ice-cold 0.4 M HClO_4_ was added and the plate was frozen at –80°C. After 24 h, the plate thawed on ice and froze again. After thawing, the supernatant was aspirated and 3 M K_3_PO_4_ was added to obtain a pH of 6.5. All samples were centrifuged (20,800 × g, 4°C, 10 min), and the supernatants were used for the analysis of nucleotide concentrations with RP-HPLC as described above. The results were expressed as nmol/mg of protein.

### Statistical analysis

Statistical analysis was performed using InStat software (GraphPad, San Diego, CA, USA). First, the normality of data distribution was assessed using the Shapiro–Wilk test. Then, comparisons of mean values were evaluated and parametrical on non-parametrical tests were used followed by *post hoc* tests as described in figure legends. The exact value of n was provided for each type of experiment. Statistical significance was assumed at *p* < 0.05.

## Results

**FIGURE 1:**
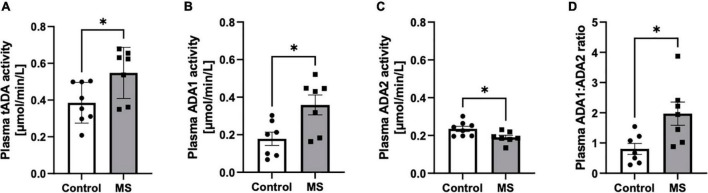
Soluble total adenosine deaminase (ADA) activity was increased in the plasma of MS patients compared to healthy subjects but individual ADA iso-enzymes displayed activity changes in opposite directions. Plasma total soluble adenosine deaminase (tADA) **(A)**, ADA1 **(B)** and ADA2 **(C)** activities and ADA1 to ADA2 ratio **(D)** in patients with multiple sclerosis (*n* = 7) and healthy controls (*n* = 8). Results are shown as mean ± SEM; *p < 0.05 by Student *t*-test **(A–D)**; ns, not significant.

**FIGURE 2:**
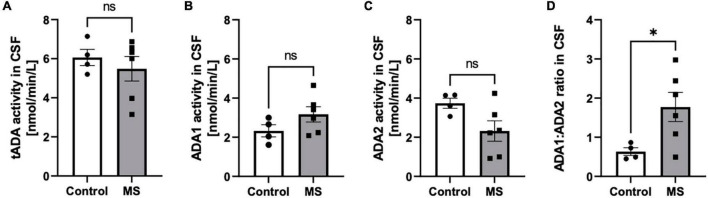
The ratio of soluble adenosine deaminase ADA1 to ADA2 activity was increased in the CSF of MS patients compared to healthy subjects. Cerebrospinal fluid (CSF) total soluble adenosine deaminase (tADA) **(A)**, ADA1 **(B)** and ADA2 **(C)** activities and ADA1 to ADA2 ratio **(D)** in patients with multiple sclerosis (*n* = 5) and healthy controls (*n* = 4). Results are shown as mean ± SEM; *p < 0.05 by Student *t*-test **(D)**; ns, not significant.

The increase of total activity of both ADA isoenzymes (tADA) in plasma of MS patients compared to healthy subjects (Figure 1A) resulted from enhanced ADA1 activity (Figure 1B), whereas no significant differences were observed in ADA2 activity (Figure 1C). The ratio of ADA1 to ADA2 was higher in the plasma of MS patients than in the control group (Figure 1D). While we did not observe any significant differences between activities of tADA (Figure 2A) and ADA1 (Figure 2B) or ADA2 (Figure 2C) alone in the CSF between MS patients and the control group, the ratio of ADA1:ADA2 was increased in the MS group (Figure 2D).

**FIGURE 3:**
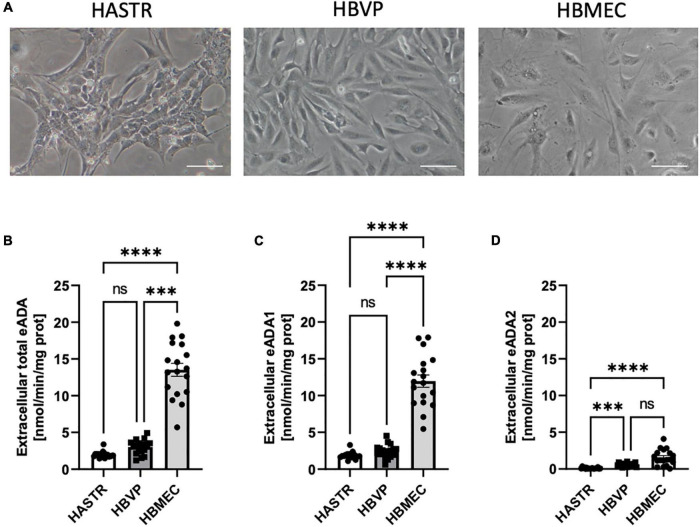
The activity of both cell-surface ecto-adenosine deaminase (eADA) iso-enzymes was highest in the brain microvascular endothelial cells. Representative images of the human brain astrocytes (HASTR), human vascular pericytes (HBVP) and human brain microvascular endothelial cells (HBMECs), scale bar = 50 μm **(A)**. The activities of cell-surface total eADA **(B)**, eADA1 **(C)** and eADA2 **(D)** in HASTR, HBVP and HBMEC **(B)**. Results are shown as mean ± SEM, *N* = 3 independent experiments; *n* = 6 biological replicates per experiment; ^***^p < 0.001, ^****^p < 0.0001 by One-way ANOVA followed by Kruskal-Wallis *post-hoc* test (*n* = **B–D**); ns, not significant.

In the single-cell culture, we observed the highest activity of total cell-surface eADA in HBMEC as compared to human brain vascular pericytes (HBVP) and human astrocytes (HASTR) (Figures 3A,B). Pericytes and endothelial cells expressed both eADA1 and eADA2, while astrocytes showed only slight activity of ADA1 (Figures 3C,D).

**FIGURE 4:**
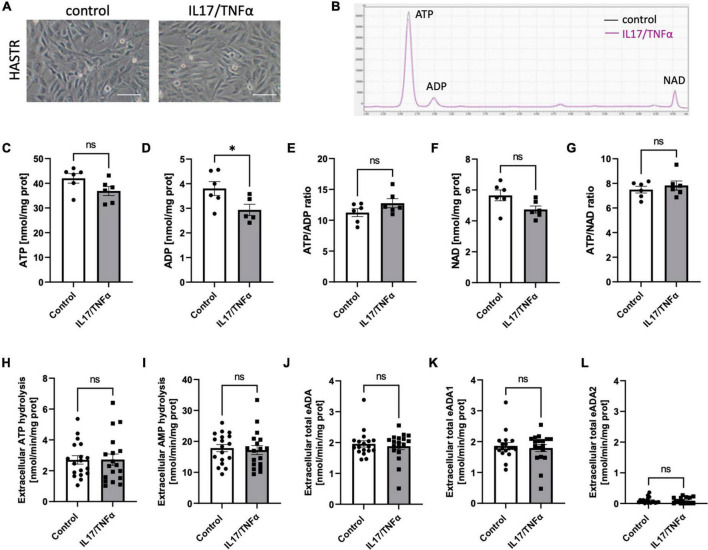
Treatment of HASTR with IL17 and TNFα did not affect cell-surface ecto-adenosine deaminase (eADA) activity. Representative images of human astrocytes treated for 18 h with 50 ng/ml IL17 and 10 ng/ml TNFα, scale bar = 50 μm **(A)**. Representative chromatogram with signals for adenosine triphosphate (ATP), adenosine diphosphate (ADP), and nicotinamide adenine dinucleotide (NAD) in control (black) and IL17/TNFα-treated (pink) astrocytes **(B)**. Intracellular ATP **(C)** and ADP **(D)** concentration and ATP/ADP ratio **(E)**, NAD **(F)** concentration and ATP/NAD ratio **(G)** in IL17/TNFα-treated astrocytes. The rate of cell-surface ATP **(H)** and AMP **(I)** hydrolysis and the activity of cell-surface total eADA **(J)**, eADA1 **(K)** and eADA2 **(L)** in IL17/TNFα-treated astrocytes **(C)**. Results are shown as mean ± SEM; *N* = 3 independent experiments; *n* = 2 **(C–G)**, *n* = 6 **(H–L)** biological replicates per experiment; *p < 0.05 by Student *t*-test; ns, not significant.

**FIGURE 5:**
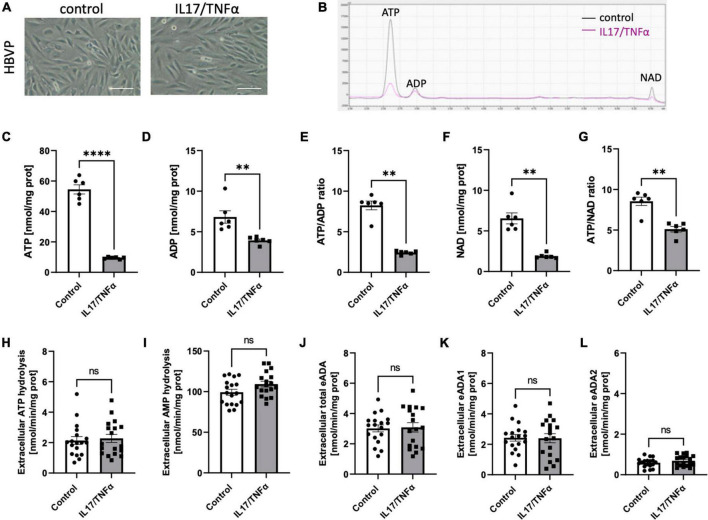
Treatment of HBVP with IL17 and TNFα did not affect cell-surface ecto-adenosine deaminase (eADA) activity. Representative images of human brain vascular pericytes treated for 18 h with 50 ng/ml IL17 and 10 ng/ml TNFα, scale bar = 50 μm **(A)**. Representative chromatogram with signals for adenosine triphosphate (ATP), adenosine diphosphate (ADP), and nicotinamide adenine dinucleotide (NAD) in control (black) and IL17/TNFα-treated (pink) pericytes **(B)**. Intracellular ATP **(C)** and ADP **(D)** concentration and ATP/ADP ratio **(E)**, NAD **(F)** concentration and ATP/NAD ratio **(G)** in IL17/TNFα-treated pericytes. The rate of cell-surface ATP **(H)** and AMP **(I)** hydrolysis and the activity of cell-surface total eADA **(J)**, eADA1 **(K)** and eADA2 **(L)** in IL17/TNFα-treated pericytes. Results are shown as mean ± SEM; *N* = 3 independent experiments; *n* = 2 **(C–G)**, *n* = 6 **(H–L)** biological replicates per experiment; ^**^p < 0.01; ^****^p < 0.0001 by Student *t*-test **(C,D)** and Mann-Whitney *U*-test **(E–G)** ns, not significant.

**FIGURE 6:**
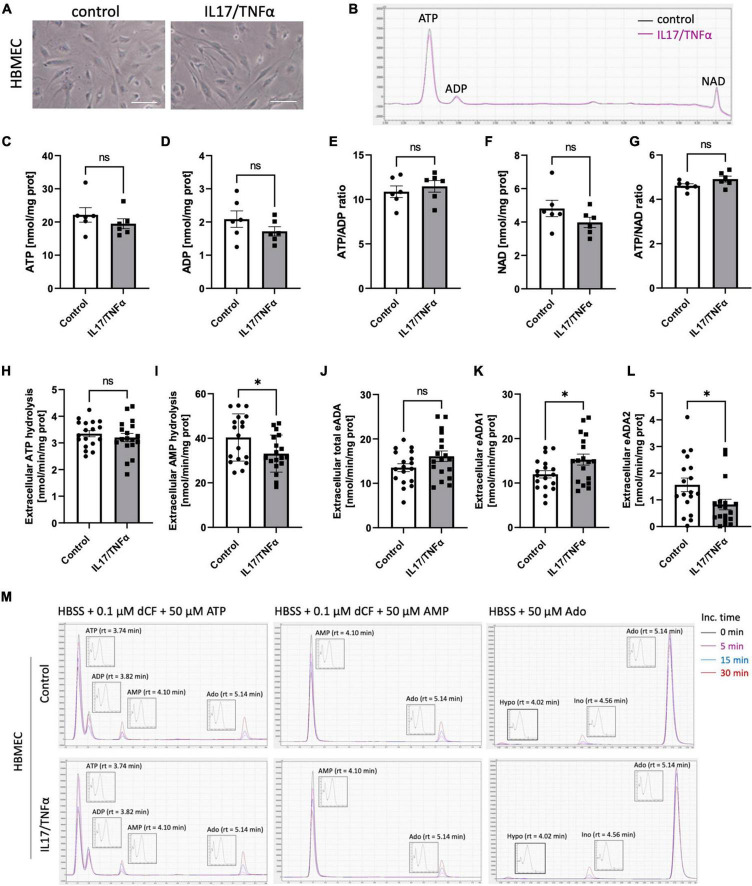
Treatment of HBMEC cells with IL17 and TNFα induced eADA1 and decreased eADA2 activities. Representative images of human brain microvascular endothelial cells (HBMEC) treated for 18 h with 50 ng/ml IL17 and 10 ng/ml TNFα, scale bar = 50 μm **(A)**. Representative chromatogram with signals for adenosine triphosphate (ATP), adenosine diphosphate (ADP), and nicotinamide adenine dinucleotide (NAD) in control (black) and IL17/TNFα-treated (pink) HBMEC **(B)**. Intracellular ATP **(C)** and ADP **(D)** concentration, ATP/ADP ratio **(E)**, NAD concentration **(F)** and ATP/NAD ratio **(G)** in IL17/TNFα-treated HBMEC. The rate of cell-surface ATP **(H)** and AMP **(I)** hydrolysis, the activity of cell-surface total ecto-adenosine deaminase (eADA) **(J)**, eADA1 **(K)** and eADA2 **(L)** in IL17/TNFα-treated HBMEC. Representative chromatograms with signals for the determination of ATP hydrolysis, AMP hydrolysis and total adenosine deamination in control and IL17/TNFα-treated HBMEC **(M)**. Results are shown as mean ± SEM; *N* = 3 independent experiments; *n* = 2 **(C–G)**, *n* = 6 **(H–L)** biological replicates per experiment; *p < 0.05 by Student *t*-test **(H,J)** or Mann-Whitney *U*-test **(K)**. ns, not significant; Inc. time, incubation time; AMP, adenosine monophosphate; Ado, adenosine; Ino, inosine; Hypo, hypoxanthine; rt, retention time.

Then, we analyzed the effects of inflammatory stimulation by IL17/TNFα on the BBB cells’ intracellular nucleotide status and cell-surface nucleotide-converting activities that included eADA. Treatment with IL17/TNFα did not change cell morphology (Figures 4A–G) and only slightly affected adenine nucleotide levels (Figures 4C–G). The rates of extracellular ATP and AMP hydrolysis, as well as adenosine deamination, were not affected by IL17/TNFα treatment of astrocytes (Figures 4H–L). In turn, the intracellular concentration of ATP, ADP and NAD as well as ATP/ADP and ATP/NAD ratios were decreased in IL17/TNFα-treated pericytes (Figures 5A–G). Despite that, in these conditions, we did not observe any changes in cell-surface nucleotide hydrolysis and adenosine deamination (Figures 5H–L). In contrast, endothelial cells after IL17/TNFα treatment showed no changes in intracellular nucleotides (Figures 6A–G). Extracellular ATP hydrolysis was either not affected (Figure 6H), but the rate of AMP hydrolysis was higher in IL17/TNFα-treated endothelial cells (Figure 6I). The activity of total eADA tended to be higher after the treatment (Figure 6J). Significant differences were also observed in ADA isoenzymes. eADA1 activity was increased (Figure 6K), while eADA2 decreased (Figure 6L) after IL17/TNFα stimulation of endothelial cells. Representative chromatograms for the analyses of nucleotide and adenosine conversions on the surface of endothelial cells are shown in Figure 6M.

## Discussion

**FIGURE 7:**
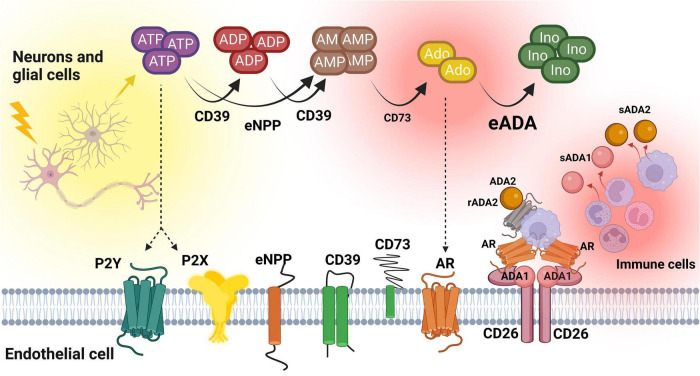
The concept of extracellular nucleotide and adenosine metabolism in multiple sclerosis. ATP, adenosine triphosphate; ADP, adenosine diphosphate; AMP, adenosine monophosphate; Ado, adenosine; Ino, inosine; CD39, ecto-nucleoside triphosphate diphosphohydrolase 1; CD73, ecto-5’-nucleotidase; eNPP, ecto-pyrophosphatase/phosphodiesterase; eADA, ecto-adenosine deaminase; ADA1, adenosine deaminase 1; ADA2, adenosine deaminase 2; sADA1, soluble adenosine deaminase 1; sADA2, soluble adenosine deaminase 2; AR, adenosine receptors; CD26, adenosine deaminase complexing protein 2/cluster of differentiation 26; P2X, purinergic 2X receptor subtype, P2Y, purinergic 2Y receptor subtype. Created with BioRender.com.

This study for the first time revealed the alterations in ADA isoenzymes’ activities in patients with MS. In both plasma and CSF, the increased ratio of ADA1 to ADA2 was observed, even though total ADA activity in the CSF was 100 times lower than in the plasma. Soluble ADA1 was significantly higher in MS patients’ plasma and tended to be higher in the CSF. The activity of ADA2 was diminished especially in the CSF. HBMEC, human brain vascular pericytes and human astrocytes revealed on their surfaces the activities of nucleotide and adenosine-converting ecto-enzymes. Under inflammatory stimulation with IL17/TNFα, we observed a lower rate of AMP hydrolysis and higher activity of eADA1 in endothelial cells. This can promote an adverse pattern that removes adenosine from its extracellular signaling. Treatment with IL17/TNFα did not affect ecto-nucleotidases and ADA isoenzymes in pericytes and astrocytes, but heightened eADA1 activity. It also diminished ADA2 in endothelial cells, reflecting the same ADA isoenzyme pattern that was observed in the CSF of MS patients (Figure 7).

Burnstock (1972) proposed the concept of purinergic signaling in the CNS in 1972. Over the years, he and the others demonstrated that both ATP and its precursore adenosine are essential for cell communication and signaling as purinergic receptors are widely expressed in neurons, oligodendrocytes, microglia, astrocytes, pericytes, and microvascular endothelial cells (Zarrinmayeh and Territo, 2020; Aslam et al., 2021; Hørlyck et al., 2021). ATP can be released at synapses together with other neurotransmitters, or extra-synaptically via plethora non-lytic mechanisms including vesicular exocytosis, ATP-binding cassette (ABC) transporters, connexin hemichannels, and pannexin channels (Lohman et al., 2012). When ATP, physiologically present inside the cells at millimolar level, is extensively released to intercellular space and increases its extracellular concentration from nanomolar to micromolar, immune cells can recognize it as an injury signal (Di Virgilio et al., 2020). The increase in ATP concentration is interpreted as a danger signal by the cells and triggers innate and adaptive immune responses via interaction with P2 purinergic receptors (Junger, 2011; Domercq et al., 2013).

In MS, modulated expression of P2Y12, P2X4 and P2X7 receptors stimulates the release of proinflammatory chemokines and cytokines as well as immune cell migration and proliferation that leads to demyelination and axonal damage (Domercq et al., 2019). P2 receptor activation can be downregulated by cell-surface ecto-nucletidases that hydrolyze nucleotides to anti-inflammatory adenosine (Zimmermann et al., 2012). This adenine nucleotide derivative directly affects G protein-coupled adenosine receptors A1, A2A, A2B, and A3 on the surface of immune and brain cells having protective effects in MS (Sánchez-Gómez et al., 2013). Especially decreased signaling via A1 and A2A adenosine receptors seem to be linked to neuroinflammation and demyelination. It has been shown that chronic administration of caffeine, an A1 receptor antagonist, resulted in the augmented expression of the A1 receptor in microglia, together with a reduction in the severity of the experimental allergic encephalomyelitis (EAE), the model of MS, accompanied by neuroinflammation and demyelination (Tsutsui et al., 2004). In turn, A2A adenosine receptors are highly expressed on infiltrating immune cells inside MS plaques correlating to tissue damage (Cekic and Linden, 2016). Moreover, in murine monocytes, knock-out of the A2A receptor substantially upregulated TNF-α production, while stimulating the A2A receptor with the agonist, CGS21680 produced a significant downregulation in TNF-α production (Haskó et al., 2000; Zhang et al., 2005). These observations were confirmed by the elevated TNF-α levels in the CSF of MS patients (Selmaj et al., 1995). Furthermore, in cultured lymphocytes from untreated MS patients, the A2A receptor agonist inhibited the release of TNF-α, IL-6, IL-1β, IFN-γ, and IL-17 following the incubation with phorbol myristate acetate. This inhibitory effect of the A2A agonist was abolished by the selective antagonist SCH 442416 indicating A2A-dependent response (Vincenzi et al., 2013).

Extracellular adenosine concentration is maintained by the balance between its cell surface production from ATP by ecto-nucleotidases, degradation by eADA, and cell uptake by nucleoside transporters (Yegutkin et al., 2000; Burnstock and Ralevic, 2014). Interestingly, it has been shown that adenosine levels are reduced in the blood of MS patients (Mayne et al., 1999). Adenosine is generated from the breakdown of ATP by the activities of ecto-nucleoside triphosphate diphosphohydrolase 1 (eNTPD1, CD39), and ecto-5’nucleotidase (e5’NT, CD73), both extensively expressed in the CNS (Wang and Guidotti, 1998; Braun et al., 2000). As we have demonstrated in this study, all analyzed cells including brain microvascular endothelial cells, brain vascular pericytes, and astrocytes revealed substantial rates of extracellular ATP and AMP hydrolysis that are mainly covered by CD39 and CD73 enzymatic activities. It has been shown before that CD73 expression on brain endothelial cells regulates lymphocyte immune surveillance between the blood and the CSF. Also, treatment of primary human brain microvascular cells and astrocytes with IFN-β upregulated CD73 expression and inhibited transmigration of CD4^+^ T cells via an *in vitro* BBB model indicating that the increased expression of CD73 is protective in MS (Niemelä et al., 2008).

Our data showed that IL17/TNFα-stimulated HBMECs decreased the rate of extracellular AMP hydrolysis resulting in reduced adenosine production which, in consequence, may diminish its protective effects. We did not observe differences in ATP and AMP hydrolysis on the surface of astrocytes after IL17/TNFα stimulation, but the decreased CD73 expression has been demonstrated in astrocytes in the EAE model where it mediated the reactivity of infiltrating T cells (Zhou et al., 2019). Moreover, the basement levels of extracellular ATP hydrolysis were similar in endothelial cells, astrocytes, and pericytes, but among these cells, the rate of AMP hydrolysis was the highest in untreated pericytes. Interestingly, unlike other cells, pericytes were the most susceptible to the decrease in intracellular nucleotide pool after IL17/TNFα stimulation. Despite this, IL17/TNFα treatment did not induce any differences in extracellular adenosine metabolism on their surface. Nevertheless, as shown in previous works and this study, brain vascular pericytes treated with pro-inflammatory stimuli may be a significant source of extracellular ATP that can enter purinergic signaling pathways (Hørlyck et al., 2021; Lee et al., 2021).

The dysregulation of CD39/CD73 has also been reported in microglia, where disrupted adenosine metabolism strongly limited survival of knock-out microglia *in vitro* and reduced microglia density in the cortex of knock-out mice (Braun et al., 2000). In turn, pharmacological inhibition of adenosine membrane transporter, the equilibrative nucleoside transporter 1 (ENT1), by dipyridamole together with activation of adenosine receptors by adenosine, restored the microglial response in CD39 knock-out mice (Matyash et al., 2017).

The last component that regulates the bioavailability of adenosine for its signaling is deamination by ADA. Inside the cells, ADA plays a minor role in adenosine metabolism as most of this nucleoside is effectively phosphorylated to AMP by adenosine kinase (ADK) that has a much lower K_m_ value for adenosine (∼1 μM) than ADA (25–150 μM) (Zhulai et al., 2022). Although, intracellular ADA is critical for maintaining a low concentration of deoxyadenosine, which, via phosphorylation to dATP, can inhibit ribonucleotide reductase (RNR) and suppress DNA synthase (Redelman et al., 1984). This provides severe immunosuppressive effects observed, for instance, in severe combined immunodeficiency (SCID) patients, who have deficient ADA activity (Kuo et al., 2020). However, precisely due to the inhibition of RNR and immunosuppressive properties, deoxynucleoside derivatives, such as cladribine (chlorodeoxyadenosine, CdA), became disease-modifying therapies for MS (Giovannoni, 2017). While CdA is a successful drug for relapsing MS, it is ineffective in some cases (Deeks, 2018; Brochet et al., 2022). Initially, it was thought that CdA is completely resistant to deamination and can be fully converted to CdATP, inhibiting RNR and further lymphocyte proliferation (Johnston, 2011). However, it was later demonstrated that CdA can be transformed into many non-active derivatives, including chlorodeoxyinosine by ADA and chloroadenine by purine nucleoside phosphorylase (PNP), which is further deaminated to chlorohypoxanthine, also by ADA (Scheible et al., 2013). Interestingly, an analysis of short nucleotide polymorphisms (SNP) of the ADA gene in a group of 561 MS patients revealed that ADA SNP rs244072 was related to increased CSF levels of TNF-α, IL-5, and RANTES and decreased levels of IL-10 (Bassi et al., 2020). Moreover, the presence of the C allele was associated with a tendency of increased lymphocyte count that, most probably, was related to an increased ADA activity in those patients, as lymphocytes are a key source of soluble, intracellular, and cell-surface ADA activity (Kutryb-Zajac et al., 2019). This can have serious implications for the use of CdA treatment in MS and, as such, a selective targeting of the ADA pathway could be more effective in the treatment of MS.

Besides significant expression of ADA in the immune cells, we observed its abundant activity on the surface of brain microvascular endothelial cells that was much more effective than in astrocytes and brain vascular pericytes. Interestingly, the stimulation with IL17/TNFα only slightly affected the total adenosine deamination rate, but significantly increased eADA1 activity and decreased eADA2. This finding sheds new light on the importance of cell surface eADA in MS pathogenesis. The greater amounts of soluble ADA that we observed in the plasma and the CSF of MS patients testify to a phenotype that reduces protective adenosine receptor signaling. This can be the effect of ADA release from ADA-rich immune cells during neuroinflammation. Furthermore, deregulated cell surface eADA1 to eADA2 ratio in brain microvascular endothelial cells that originated from the enhanced eADA1 and diminished eADA2 reflects soluble ADA isoenzyme pattern in the CSF. This may be due to the possible shedding of eADA isoenzymes from the surface of endothelial cells. On the other hand, both ADA1 and ADA2 have extra-enzymatic properties that can play a role in MS (Zavialov and Engström, 2005; Franco et al., 2007). ADA1 by the formation of trimeric complexes with CD26 protein and adenosine receptors may facilitate binding of lymphocytes to endothelial cells triggering neuroinflammation (Moreno et al., 2018). Whereas decreased ADA2 activity as a growth factor for M2-polarized macrophages can redirect them, and the microglial cells, to the M1 pro-inflammatory phenotype that, at early stages of MS, leads to severe tissue damage in the CNS (Chu et al., 2018; Watanabe et al., 2019; Kutryb-Zajac et al., 2021). Therefore both ADA isoenzymes may serve as therapeutic targets for MS with the potential to decrease the adhesion mode exhibited by ADA1, inhibit their catalytic activity, or induce the growth factor properties of ADA2 by, for example, the recently proposed pegylated-ADA2 (PEG-ADA2) treatment (Wang et al., 2021).

## Conclusion

In conclusion, the data here presented indicates that the increased ADA1 to ADA2 ratio in MS CSF and plasma may translate to an unfavorable phenotype that triggers ADA1-mediated pro-inflammatory mechanisms and decreases the ADA2-dependent neuroprotective and growth-promoting effects.

## Data availability statement

The original contributions presented in this study are included in the article/supplementary materials, further inquiries can be directed to the corresponding author/s.

## Ethics statement

The studies involving human participants were reviewed and approved by the Independent Bioethics Committee For Scientific Research at the Medical University of Gdańsk, Poland under the license number: NKBBN/457/2019. The patients/participants provided their written informed consent to participate in this study.

## Author contributions

BK-Z and AR: conceptualization, supervision, project administration, and funding acquisition. AK, BK-Z, FC, KU, AB, TF, and AR: methodology. AK, BK-Z, and KU: software. BK-Z, KU, and AK: validation. AK, BK-Z, FC, KU, and AR: investigation. BK-Z and KU: data curation. TF, BK, and AR: resources. AK, BK-Z, and AR: writing—original draft preparation. BK and RS: writing—review and editing. BK-Z: visualization. All authors have read and agreed to the published version of the manuscript.
